# The different influences of drought stress at the flowering stage on rice physiological traits, grain yield, and quality

**DOI:** 10.1038/s41598-019-40161-0

**Published:** 2019-03-06

**Authors:** Xiaolong Yang, Benfu Wang, Liang Chen, Ping Li, Cougui Cao

**Affiliations:** 1 College of Plant Science and Technology, Huazhong Agricultural University, Wuhan, Hubei 430070 China; 2 Hubei Collaborative Innovation Center for Grain Industry, Yangtze University, Jingzhou, Hubei 434025 China

## Abstract

Seasonal drought is a major threat to rice production. However, the sensitivity of rice to drought stress (DS) at different growth periods remains unclear. The objective of this study was to reveal the different impacts of DS at the flowering stage on rice physiological traits, grain yield, and quality. Field experiments were conducted with two rice cultivars, Yangliangyou 6 (YLY6) and Hanyou 113 (HY113) under two water treatments (traditional flooding (CK) and DS at flowering stage) in 2013 and 2014. Compared with CK, grain yield (GY) under DS was significantly reduced by 23.2% for YLY6 and 24.0% for HY113 while instantaneous water use efficiency (IWUE) was significantly increased by 39% for YLY6 and 37% for HY113, respectively. All physiological traits were significantly decreased under DS and physiological activities did not revert to normal levels at grain filling stage. There was no significant effect on the appearance and nutritional quality except for the significant increase in chalky kernel and chalkiness under DS. Our data suggest that drought stress at flowering stage has a strong influence on rice physiological traits and yield. Stronger recovery capability contributes to maintaining relatively high grain production, which could be a great target for the breeder in developing drought-tolerant rice cultivars.

## Introduction

Rice (*Oryza sativa* L.) is a staple food for nearly half of the world,s population, and its production is urgent needed with the rapidly increase of population in coming decades, especially in Asia countries^1,2^. Rice consumes almost 80% of the total irrigation freshwater resources^3,4^. However, with urbanization and industrialization, fresh water is becoming rare, DS may be a great challenge to agricultural production all over the world^5^. According to statistics, approximately 42 million hectares of rice is subject to occasional or frequent DS in Asia, resulting in significant yield loss^6^. Thus, it is important to provide more staple rice to keep global food security and to meet the food needs of a growing world population^7^. As the major rice production region, South China produces more than 80% of cereal food of the country^8^. Although abundant rainfall is supplied in South China, water shortage is still exist because of the uneven distribution of among seasons and regions^9^. Therefore, it is important to save water and increase the crop water use efficiency (WUE) in arid, semi-arid areas and seasonal drought regions^4,10^.

Rice is highly susceptible to water stress during the reproductive stage, leading to significant reduction in grain GY^11,12^. The yield loss magnitude depends on the growth stage and duration, the severity of DS^13,14^. Severe DS applied at the vegetative stage and mild DS applied at the flowering stage in rice in one trial result in 20% and 28% yield loss, respectively^15^. However, the field experiment of major rice varieties remains unclear in Hubei Province, China. Besides the yield of rice, grain quality is an important indicator for farmers, the milling, appearance and nutrient characters of grain were determined both environmentally and genetically^16,17^. Grain quality of rice including the percentages of brown rice rate (BRR), milled rice rate (MRR), head rice rate (HRR) in total rice grains, chalky kernel (CHK) and chalkiness (CH), the grain shape (GS) of length and width, protein content, amylose content and alkali spreading value is very important in agriculture^17–19^. All quality parameters were measured according to Rice Quality Measurement Standards (Ministry of Agriculture PR China, 1988)^16,20^. Generally, BRR, HRR, and MRR in total rice grains, CH and CHK have negative effects on grain quality. Thus, protein content, amylose content might have positive effects^21,22^. Flowering stage is critical for rice yield and quality formation. Thus, it is significant to study different influences of DS at the flowering stage on rice physiological traits, GY, and quality and to reveal the influence mechanism. Although some studies have studied GY and quality under different cultivation conditions and cultivars^1,2,17^, the mechanisms of crops respond to DS at flowering stage remains largely unknown, affecting the improvement of drought-tolerant crops^23^.

In addition, improvement of WUE for securing the environmental sustainability of rice production in southern seasonal drought areas, since rice production relies on the use of large volumes of fresh water^24^. Generally, WUE is determined by the ratio of GY to total water consumption (rainfall and irrigation) during the whole growth period^17,25^, by the ratio of photosynthetic rate (P_n_) to transpiration rate (T_r_)^26^. Moreover, carbon isotope discrimination (Δ) as an indirect mean to explain WUE is widely used in wheat^27^, rice, and there may be negative relationship between WUE and Δ^22,28,29^. Thus, WUE is important to crop yield under DS^30,31^, and it can be calculated with the ratio of yield to irrigation^17^, P_n_ to T_r_^26^ and Δ^32^.

Rice GY is closely related to photosynthesis of the functional leaves, solar energy and atmospheric carbon dioxide (CO_2_) captured by crops through photosynthesis is the main component of yield^33–35^. Therefore, it is necessary to understand the mechanisms of leaf gas exchange, photosynthesis and other related physiological traits to DS^36^. Rice production is limited by water availability and the low leaf-level photosynthetic capacity of many cultivars^20^. Generally, the decrease of stomatal conductance (G_s_) in leaves is accompanied with reducing transpiration rate, resulting in the loss of photosynthesis and lower production in biomass^17,37^. As an important indicator of drought resistance in rice, higher leaf water potential (LWP) is considered as the anti-dehydration mechanism, and the decrease of leaf-level P_n_ is always accompanied with LWP decrease and relative water content^38,39^. In addition, a long-term soil water deficit may change the rice canopy microclimate, but an intense physiological activity under a lower organ temperature was observed, indicating that a relatively lower canopy temperature or organ temperature could enhance resistance to heat and stress injury^40^. Actually, the transpiration rate could decrease under water deficit, leading to the decrease of energy consumption for transpiration and the air-leaf temperature difference^41^. In the last two decades, more frequent seasonal drought struck southern of China, causing water crisis for residents and damaged millions of hectares croplands^42,43^. Reduction in rice GY by DS occurs during reproductive development, especially at the flowering stage, and mild DS may result in severe GY loss^6,44^. The objectives of our study were to reveal the impact of DS at the flowering stage on rice yield and quality performance and to elucidate the physiological response to drought underlying the changes of yield and quality. The P_n_, G_s_, T_r_, instantaneous WUE (IWUE), Δ, LWP, air-leaf temperature gap (ALTG), GY and grain quality were determined in this study. To better understand the mechanism of change in spikelets sterility, this study focused on the physiological changes of the flag leaves of rice, particular in LWP and ALTG under DS at the flowering stage.

## Results

### Analysis of variance

In this study, field experiments were conducted with YLY6 and HY113 in 2013 and 2014 in Wuhan, and the detailed information of whole growing season was shown in Table 1. Generaly, the maximum temperature, minimum temperature, and average temperature showed no difference between 2013 and 2014, the sunshine and precipitation were significantly higher in 2013 than in 2014. Variation in the precipitation across months was greater in 2013 than in 2014, and low precipitation was observed in August 2013 and in June 2014. Analysis of variance (F-values) for GY, grain components and some physiological traits of rice among years, varieties and treatments were shown in Table 2. Significant differences (*P* < 0.05) were occurred for most evaluated traits between the two varieties and among treatments.

**Table 1:** Mean temperature, maximum temperature, minimum temperature, sunshine and precipitation per month during the whole growing season of rice across two years (2013–2014) in Wuhan, China.

Year	Month	Average temperature (°C)	Maximum temperature (°C)	Minimum temperature (°C)	Precipitation (mm per month)	Sunshine (h per month)
2013	May	23.0	34.0	15.0	206	146
	Jun	26.3	38.1	18.3	205	207
	Jul	30.6	38.0	23.6	479	280
	Aug	31.1	40.4	23.9	25.3	233
	Sep	23.7	34.1	14.3	201	149
	Oct	20.2	31.8	9.8	3.80	235
Mean		25.8	36.1	17.5	187	208
2014	May	22.1	32.1	13.8	163	157
	Jun	26.0	33.8	21.9	46	113
	Jul	27.6	37.6	20.8	134	181
	Aug	26.2	37.0	20.5	115	115
	Sep	24.5	33.8	17.8	64	133
	Oct	20.3	30.4	13.1	144	206
Mean		24.4	34.1	18.0	111	151

**Table 2:** Analysis of variance (F-values) for GY, grain components and some physiological traits of rice among years, varieties and treatments.

Source of variation	df	GY	SPN	FG	CH	LWP	ALTG	P_n_	G_s_	T_r_	IWUE	Δ
Year (Y)	1	0.08 ns	0.05 ns	0.02 ns	20.5**	748**	12.6**	1.52 ns	4.20 ns	8.38*	2.19 ns	5.01*
Varieties (V)	1	3.33 ns	12.2**	5.81*	248**	227**	68.5**	1.56 ns	1.09 ns	63.0**	28.5**	100**
Treatment (T)	1	352**	137**	159**	230**	469**	574**	142**	176**	509**	119**	1.13 ns
Y × V	1	19.9**	0.79 ns	3.40 ns	9.21 ns	319**	83.5**	2.01 ns	0.37 ns	22.7**	3.82 ns	1.68 ns
Y × T	1	15.5**	0.15 ns	10.6**	14.9**	80.5**	75.8**	0.16 ns	1.26 ns	0.08 ns	2.42 ns	19.0**
V × T	1	1.95 ns	1.66 ns	0.50 ns	52.0**	9.83**	45.8**	0.37 ns	4.52 ns	3.47 ns	0.29 ns	3.13 ns

Grain yield, GY; Spikelets per panicle, SPN; Filled grains, FG; Chalkiness, CH; Leaf water potential, LWP; Air-leave temperature gap, ALTG; Net photosynthetic, P_n_,; Stomatal conductance, G_s_; Instantaneous water use efficiency, IWUE; Carbon isotope discrimination, Δ.

*Significant at the 0.05 probability level.

**Significant at the 0.01 probability level.

### GY, yield components, and grain quality

DS at flowering stage significantly reduced GY for both YLY6 and HY113. No significant difference in GY occurred between two rice genotypes (Table 3). Yield components of the number of spikelets per panicle (SPN) and filled grains (FG) were significantly reduced by 18%, and 19% for YLY6 and 21%, and 19% for HY113, respectively, when compared with the traditional flooding irrigation system on average across two seasons, which contribute to the significantly decreased in GY in YLY6 and HY113 and reduced by 23.2%, 24.0% respectively under DS. In addition, DS at flowering stage had no significant effect on the effective panicle (EP) and 1000-grain weight (TGW).

**Table 3:** GY and yield components of two rice cultivars of YLY6 and HY113 under DS at flowering stage in 2013 and 2014.

Year	Varieties	Treatment	SPN	EP (×10^3^ m^−2^)	FG (%)	TGW (g)	GY (t.ha^−1^)
	YLY6	CK	164 a	43.5 a	75.5 a	26.2 a	10.45 a
2013		DS	139 b	34.5 b	61.2 b	25.7 a	8.03 b
	HY113	CK	180 a	43.3 a	85.3 a	27.1 a	10.65 a
		DS	144 b	35.1 b	67.4 b	26.8 a	8.09 b
	Mean		156	39.1	72.4	26.5	9.31
	YLY6	CK	166 a	40.9 a	77.8 a	26.3 a	10.35 a
2014		DS	131 b	29.4 b	63.4 b	25.8 a	9.12 b
	HY113	CK	178 a	43.4 a	74.8 a	27.1 a	10.06 a
		DS	139 b	32.9 b	61.7 b	27.2 a	8.39 b
	Mean		153	36.7	69.4	26.6	9.48

Different letters indicate statistical significance according to LSD *(P* = 0.05). TGW and GY are expressed at 14% moisture content.

No significant differences existed in BRR, MRR, HRR and GS for YLY6 and HY113, and DS at the flowering cause no significant change for these traits either (Table 4). Compared with CK, CHK and CH under DS were significantly increased by 67%, 74% in YLY6 and 53%, 76% in HY113 on average across two seasons, respectively. When the comparison was made between YLY6 and HY113 under the same water management, the head rice rate of YLY6 was 13% higher than that of HY113 under CK and 20% higher under DS; and CHK and CH degree of HY113 were higher than YLY6, CHK of HY113 was 122% higher under CK, 99.2% higher under DS than that of YLY6, respectively, CH of HY113 was 81% higher under CK, 82% higher under DS than that of YLY6, respectively.

**Table 4:** Milling and appearance quality of two rice cultivars of YLY6 and HY113 under DS during flowering stage in 2013 and 2014.

Year	Varieties	Treatment	BRR (%)	MRR (%)	HRR (%)	CHK (%)	CH (%)	GS (%)
	YLY6	CK	79.4 a	69.3 a	59.9 a	15.1 d	4.10 c	2.90 a
2013		DS	79.9 a	69.9 a	62.6 a	25.5 c	8.40 b	2.90 a
	HY113	CK	77.7 a	66.6 a	53.4 b	37.0 b	9.00 b	2.90 a
		DS	77.8 a	66.0 a	50.2 b	58.9 a	15.6 a	2.90 a
	YLY6	CK	82.1 a	71.7 a	67.6 a	15.1 d	5.63 c	2.83 b
2014		DS	81.1 a	70.9 a	67.3 a	24.9 c	8.07 ab	2.77 b
	HY113	CK	81.4 a	71.0 a	59.8 b	30.0 b	7.97 ab	3.00 a
		DS	80.8 a	70.3 a	59.1 b	44.0 a	14.2 a	3.00 a

Different letters indicate statistical significance according to LSD (*P* = 0.05).

Under CK and DS conditions, the protein content, amylose content and alkali spreading value of every variety showed no significant difference. However, the two rice varieties displayed significant difference in protein content under both CK and DS conditions. Generally, YLY6 showed 25% and 27% higher protein content than HY113 under CK and DS conditions, respectively (Table 5).

**Table 5:** Nutritional quality of two rice cultivars of YLY6 and HY113 under DS at flowering stage in 2013 and 2014.

Year	Varieties	Treatment	Protein content (%)	Amylose content (%)	Alkali spreading value
	YLY6	CK	11.0 a	16.9 a	7.23 a
2013		DS	11.1 a	17.1 a	7.30 a
	HY113	CK	8.77 b	16.8 a	7.17 a
		DS	8.70 b	17.1 a	7.17 a
	YLY6	CK	10.5 a	17.1 a	7.13 a
2014		DS	10.7 a	17.1 a	7.20 a
	HY113	CK	8.40 b	16.7 a	7.13 a
		DS	8.47 b	16.7 a	7.00 a

Different letters indicate statistical significance according to LSD (*P* = 0.05).

### The physiological change in response to DS

**Figure 1:**
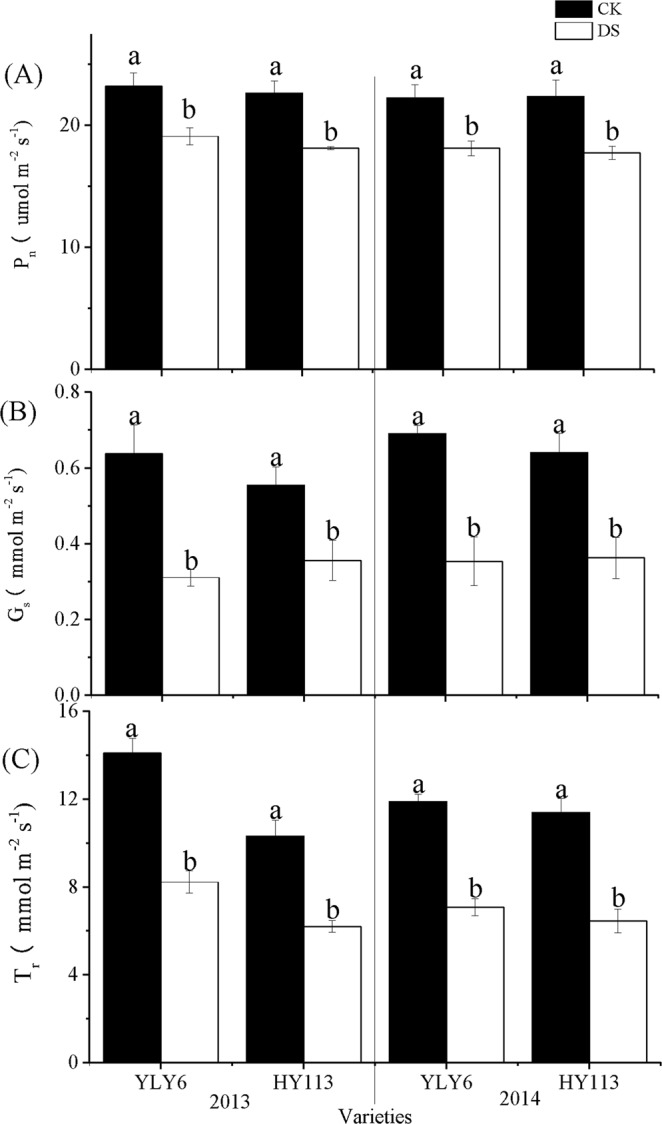
P_n_ (**A**), G_s_ (**B**), T_r_ (**C**) of two rice cultivars of YLY6 and HY113 under DS at flowering stage in 2013 and 2014. CK indicates traditional flooding cultivation and DS is drought stress at the flowering stage. The different letters in different columns under each cultivar are significantly different at the *P* = 0.05 level. Vertical bars represent standard errors.

The physiological traits including P_n_, G_s_, and T_r_ of leaves were significantly decreased under DS during the flowering stage (Fig. 1). The P_n_, G_s_, and T_r_ of leaves reduced by 24%, 41%, 30% on average across two seasons respectively. The P_n_, G_s_ of leaves showed no significant difference between YLY6 and HY113 under the same water treatment across two seasons (Fig. 1). T_r_ of YLY6 was slightly higher than that of HY113 under both water treatments, with no statistical difference.

**Figure 2:**
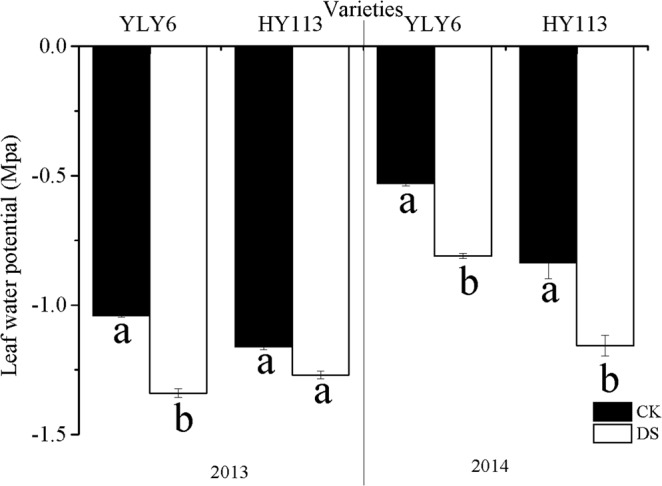
LWP of leaves of two rice cultivars of YLY6 and HY113 under DS at flowering stage in 2013 and 2014. CK indicates traditional flooding cultivation and DS is drought stress at the flowering stage. The different letters in different columns under each cultivar are significantly different at the *P* = 0.05 level. Vertical bars represent standard errors.

**Figure 3:**
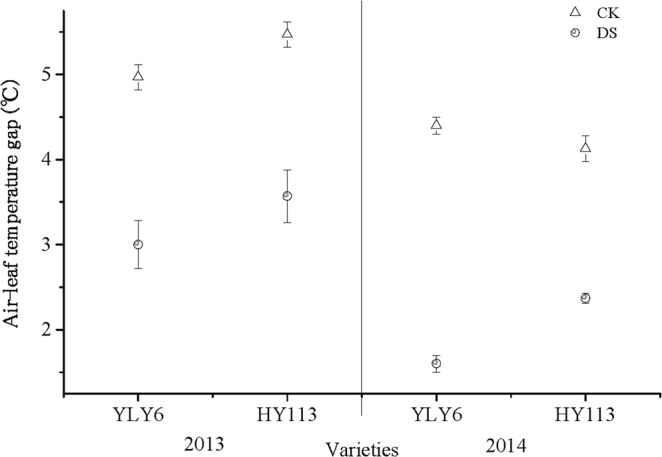
ALTG of two rice cultivars of YLY6 and HY113 under DS at flowering stage in 2013 and 2014. CK indicates traditional flooding cultivation and DS is drought stress at the flowering stage. The different letters in different columns under each cultivar are significantly different at the P = 0.05 level. Vertical bars represent standard errors.

Generally, LWP of two cultivars (YLY6, HY113) showed lower level under DS than CK in 2013 and 2014, except for HY113 in 2013 (Fig. 2). To better understand the leaf temperature responses to DS, we calculated the temperature gap between air and leaves. As shown in Fig. 3, the DS significantly decreased the ALTG when compared with CK under the similar temperature and relative humidity. The ALTG was reduced by 51% in YLY6 and 39% in HY113 on average across two seasons respectively. There was no significant difference between two cultivars YLY6 and HY113 under CK, while there was a significant increase in ALTG in HY113 compared with YLY6 under DS, the difference was 16% and 32% in 2013 and 2014, respectively.

### IWUE and Δ

**Figure 4:**
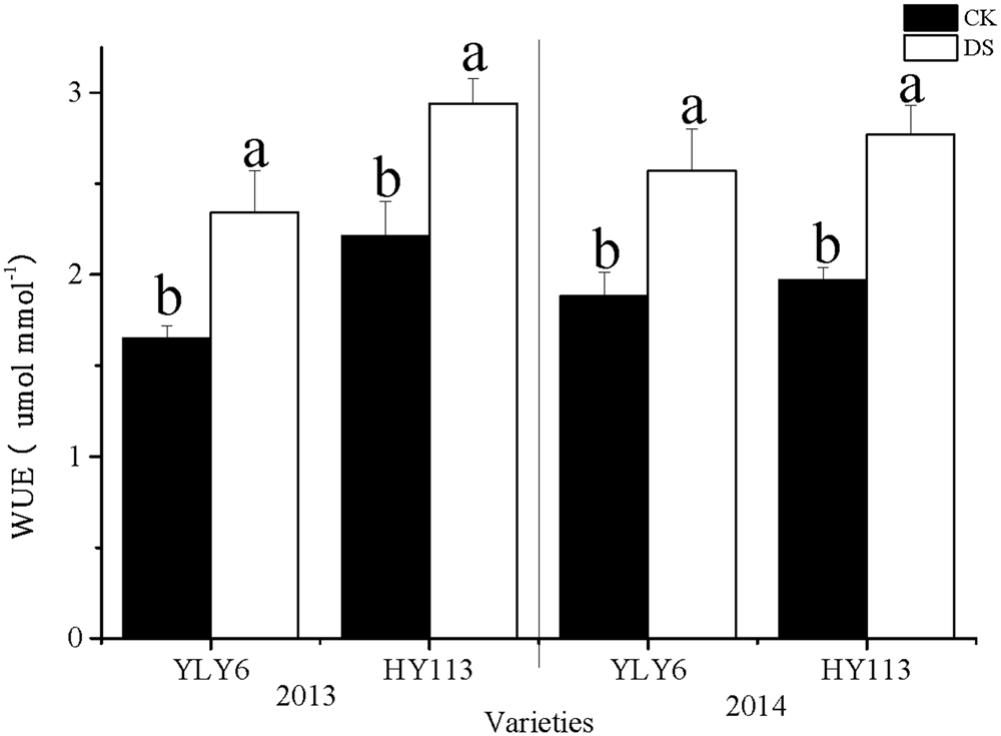
The IWUE of two rice cultivars of YLY6 and HY113 under DS at flowering stage in 2013 and 2014. CK indicates traditional flooding cultivation and DS is drought stress at the flowering stage. The different letters in different columns under each cultivar are significantly different at the P = 0.05 level. Vertical bars represent standard errors.

Compared with that under the traditional flooding cultivation, the IWUE of these two cultivars was remarkably increased under DS conditions, with 39% for YLY6, 37% for HY113 on average across two seasons (Fig. 4). Under DS, the drought tolerance cultivar HY113 showed higher IWUE than YLY6 by 17%, and the higher water productivity of HY113 might contribute to the improved drought tolerance.

**Figure 5:**
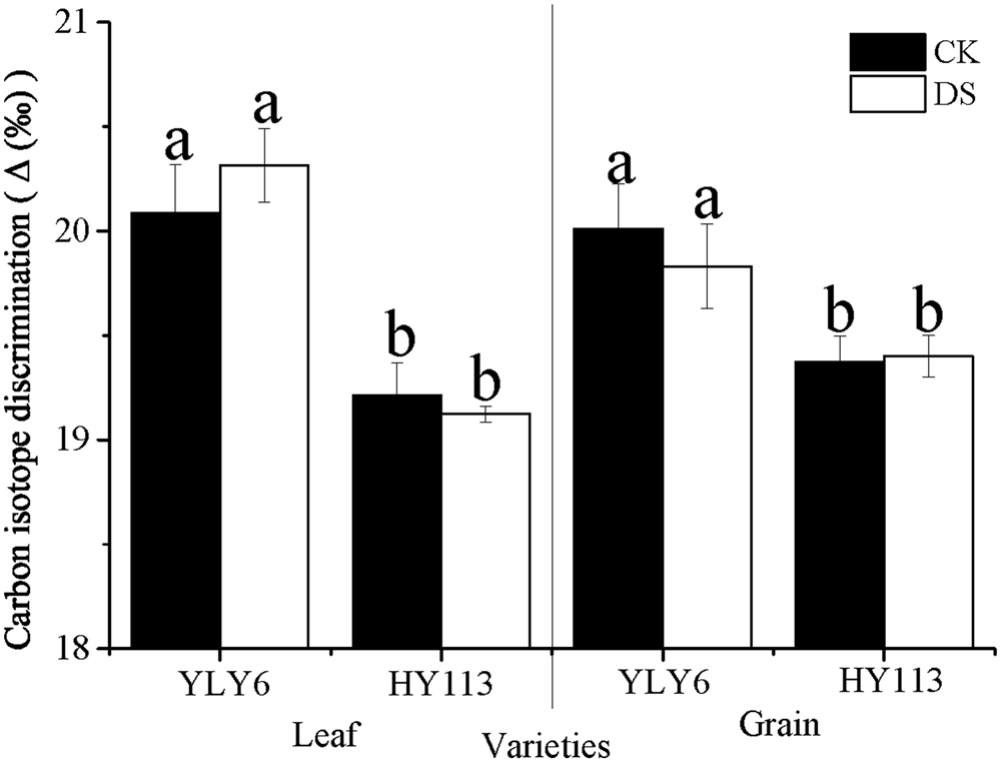
The Δ of two rice cultivars of YLY6 and HY113 under DS at flowering stage in 2014. CK indicates traditional flooding cultivation and DS is drought stress at the flowering stage. The different letters in different columns under each cultivar are significantly different at the P = 0.05 level. Vertical bars represent standard errors.

To better understand the response of WUE to DS, Δ in leaves and grain was measured. Although no significant difference between CK and DS in leaves and grain of every cultivar, YLY6 and HY113 showed significant differences in leaves and grain under both CK and DS conditions (Fig. 5).

### The process of physiological changes in response to DS

No significant difference in LWP, ALTG, P_n_, G_s_, and T_r_ occurred before DS and significant decrease were observed under DS. After 2 days of rehydration, most evaluated physiological traits could return to the normal level. However, significant decrease was observed after rehydration for 20 days compared to CK. As shown in Table 6, LWP, ALTG, P_n_, G_s_, and T_r_ were reduced by 31%, 27%, 35%, 41% and 21%, respectively. Thus, the effect of drought stress on physiological changes might be appeared at the grain filling period, and these changes might attribute to DS-triggered early senescence.

**Table 6:** The dynamic of LWP, ALTG and photosynthetic traits of YLY6 and HY113 at flowering stage in 2014.

Varieties	Treatment	LWP (Mpa)				ALTG (°C)				P_n_ (umol.m^−2^.s^−1^)				G_s_ (mmol. m^−2^.s^−1^)				T_r_ (mmol. m^−2^.s^−1^)			
		BDS	DS	ARD2	ARD 20	BDS	DS	ARD2	ARD 20	BDS	DS	ARD2	ARD 20	BDS	DS	ARD2	ARD 20	BDS	DS	ARD2	ARD 20
YLY6	CK	−0.58a	−0.53a	−0.49a	−0.62a	2.57a	4.40a	2.37a	0.90a	22.7a	22.3a	22.8a	15.5a	0.62a	0.69a	0.55a	0.18a	8.42a	11.9a	9.15a	4.64a
	DS	−0.63a	−0.81b	−0.54a	−0.84b	2.53a	1.60b	1.60b	0.50b	22.6a	18.1b	21.4a	11.9b	0.62a	0.35b	0.57a	0.12 b	8.30a	7.07b	8.09b	3.40b
HY113	CK	−095a	0.84a	−0.77a	−0.81a	4.20a	4.13a	3.23 a	1.27a	21.5a	22.4a	23.4a	15.6a	0.55a	0.64a	0.65a	0.33 a	6.77a	11.4a	9.64a	6.22a
	DS	−0.99a	−1.16b	−0.66a	−1.02b	4.07 a	2.37b	1.67 b	1.15a	21.3a	17.8b	21.5a	8.42b	0.53a	0.36b	0.55b	0.17b	6.37a	6.44b	6.50b	5.26a

Before drought stress, BDS; Drought stress, DS; After 2 days of rehydration, ARD 2; After 20 days of rehydration, ARD 20; Photosynthetic, P_n_; Stomatal conductance, G_s_; Ttranspiration rate, T_r_. Different letters indicate statistical significance according to LSD (*P* = 0.05).

### Correlation among traits for DS

The GY was positively correlated (*P* < 0.05) with SPN, FG, P_n_, G_s_, LWP and ALTG, and negatively correlated with CH and IWUE (*P* < 0.05) (Table 7); the FG was significantly positively correlated with SPN; the P_n_ was significantly positively correlated with SPN, FG, and ALTG; the G_s_ was significantly positively correlated with SPN, FG, LWP, ALTG, and P_n_; the ALTG was significantly positively correlated with SPN and FG; the IWUE was significantly negatively correlated with SPN, FG, P_n_, G_s_, LWP, and ALTG, significantly positively correlated with CH; the CH was significantly negatively correlated with FG, LWP, P_n_, and G_s_.

**Table 7:** Correlations among the physiological traits of YLY6 and HY113 under drought stress at flowering stage across two years.

	GY	SPN	FG	CH	LWP	ALTG	P_n_	G_s_	IWUE
SPN	0.717**								
FG	0.787**	0.855**							
CH	−0.581*	−0.322	−0.409						
LWP	0.567*	0.252	0.370	−0.561*					
ALTG	0.599*	0.678**	0.770**	−0.235	−0.078				
P_n_	0.729**	0.777**	0.737**	−0.625**	0.297	0.661**			
G_s_	0.785**	0.698**	0.734**	−0.535*	0.614*	0.497*	0.733**		
IWUE	−0.722**	−0.618*	−0.623*	0.832**	−0.487*	−0.467*	−0.755**	−0.747**	
Δ	0.225	−0.175	0.078	−0.620**	0.324	−0.007	0.192	0.126	−0.314

*Significant at the 0.05 probability level.

**Significant at the 0.01 probability level.

**Figure 6:**
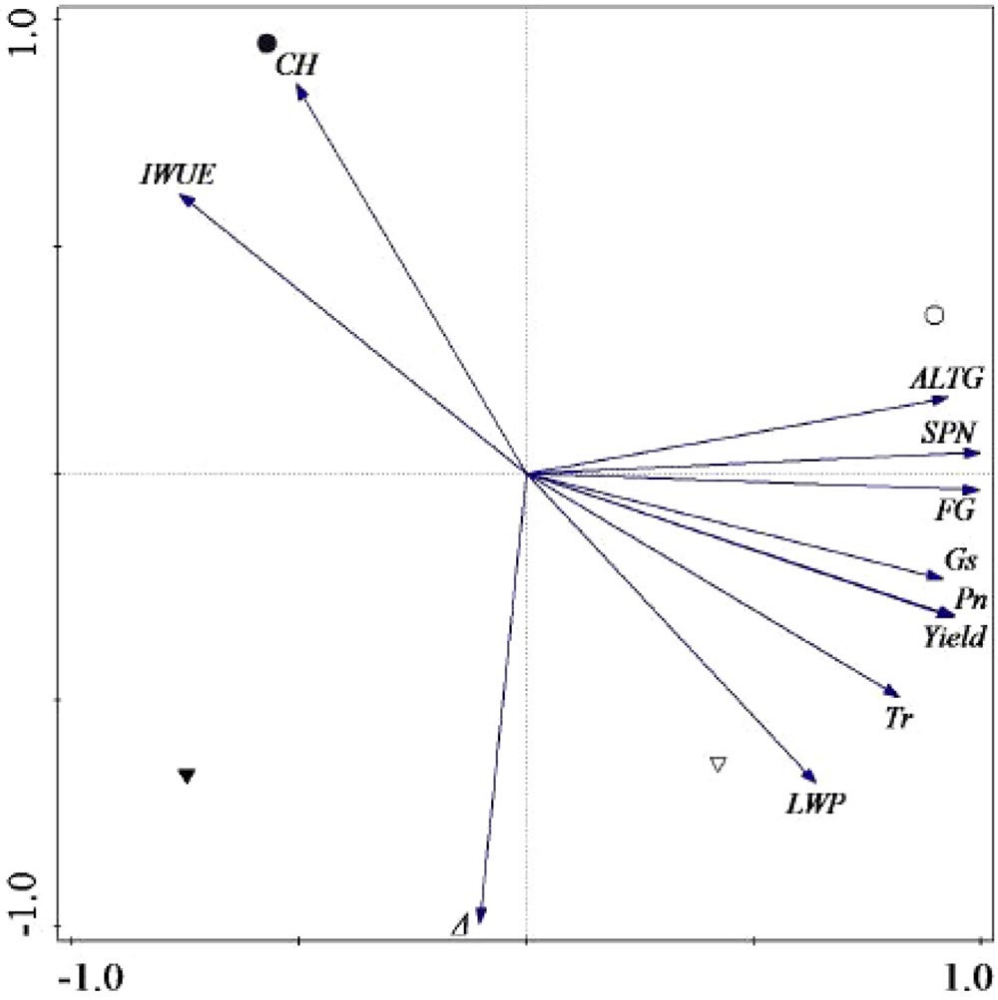
Principal component analysis of target traits. Grain yield, GY; Spikelets per panicle, SPN; Filled grains, FG; Chalkiness, CH; Leaf water potential, LWP; Air-leave temperature gap, ALTG; Net photosynthetic, P_n_,; Stomatal conductance, G_s_; Instantaneous water use efficiency, IWUE; Carbon isotope discrimination, Δ; HY113-drought stress (●); HY113-traditional flooding (◯); YLY6-drought stress (▼); YLY6- traditional flooding (∇).

As showed in the Fig. 6, the main ordination axis was significantly positively correlated with ALTG, SPN, FG, G_s_, P_n_, Yield, T_r_ and LWP at the flowering stage, while ALTG, SPN, FG had great contributions to ordination axis. HY113-DS and YLY6-DS were negative closely related to the main ordination axis, which indicates that DS had significant effects on flag leaf photosynthetic capacity. Moreover, HY113-DS was significantly positively correlated IWUE and CH.

## Discussion

The effect of DS on rice growth at different growth periods has been widely studies. Rice can be subject to DS at any time and the responses of growth are different in different growth stages^31,45,46^. Some studies have showed that the most sensitive period to DS of rice development is booting stage^31^, and controlled irrigation and drainage at vegetative stages might be the most critical period^47–50^. Other studies found that the flowering stage is the most sensitive stage to water deficit^51,52^. The formation of rice panicle and spikelets morphogenesis are primary factors of rice yield^53,54^. Consistent with previous studies^50,52,55–58^, our results showed that DS (−30 kPa) at the flowering stage had significant effects on GY, with the reduction of 23.2% in YLY6 and 24.0% in HY113, respectively (Table 2). Moreover, SPN and FG under DS conditions might have positive relation with GY based on the correlation analysis (Table 7).

When the rice was exposed to severe DS at the flowering stage, the significant increase in spikelets sterility and GY reduction were observed^38,51,56,59–61^, with the decline in SPN and FG. Reduction in LWP under DS resulted in the birth defect of panicle^62^ and a negative correlation was found between LWP and spikelet sterility at flowering stage under DS^38^ and another similar indicator relative water content was conducted to demonstrate the same variation trend^51,61,63^. Our result showed no significant correlation between SPN and LWP (Table 7), so LWP as an indicator for determining spikelets sterility needs further validation. Moreover, ALTG displayed positive relation with SPN and FG (Table 7). Previous studies^60,61^ showed that spikelet fertility is sensitive to high temperature. The significant decline in ALTG under DS (Fig. 3) indicated that rice may encounter high-temperature stress for the shelter proof in greenhouse (Table 1) and anther dehiscence might contribute to the reduction in SPN and FG^61^. Moreover, high temperature may be a limiting factor of root and microorganism activity at the reproductive stage and accelerate leaf senescence^64^. In general, rice cultivation could be subject to DS accompanied with some degree of high-temperature stress. On the other hand, P_n_ and capacity of flag leaf may be the primary source of crop yield and were susceptible to abiotic stress, especially by DS^26,35^. We observed that the P_n_, G_s_, and T_r_ were significantly reduced (Fig. 1), and significant positive correlated with yield, in agreement with previous studies. The leaf stomatal closure resulted in limitation to carbon dioxide uptake, which reduced the source size, additionally, the leaf senescence might also explain the limitation to dry conversion to panicle during milk stage^65,66^. After 20 days of rehydration, YLY6 exhibited higher LWP and P_n_ than HY113 (Table 6), indicating that these physiological activities during grain filling stage might contribute to the reduction in GY.

Herein, DS led to both yield losses and poor grain quality with the increase of CHK and CH degree, with no significant effect on TGW, MRR, HRR and GS (Table 4), protein content, amylose content and alkali spreading value (Table 5). Under CK and DS conditions, YLY6 showed 20% higher HRR and 27% higher protein content than HY113, while HY113 showed 99% higher CHK and 82% CH (Tables 4, 5). This result suggested that YLY6 could maintain nutritional quality under DS. CHK and CH are mainly determined by the capacity of photosynthate from whole growth period and variation might exist in different cultivars due to genetic effects^18^. Our results showed that there was a negative correlation between photosynthetic and CH (Table 7) and significant difference was observed between YLY6 and HY113 (Table 4), DS triggered low photosynthetic capacity might also contribute to the increased CH^67,68^, besides higher temperature after flowering stage^69,70^.

Our results showed that IWUE of YLY6 and HY113 was 39% and 37%, respectively, and HY113 showed 17% higher IWUE than YLY6 under DS at flowering stage (Fig. 4), indicating that P_n_ was less susceptible to reduction of G_s_ than T_r_ under DS. Moreover, GY of YLY6 and HY113 were reduced by 23.2% and 24.0% under DS, respectively (Table 3), and a significant negative correlation relationship was observed between GY and IWUE (Table 7; Fig. 6), the balance between higher crops water productivity and the loss of crops production caused by DS could be another concerned topic^41^. Our results showed a negative correlation between ALTG, LWP and IWUE (Table 7), a high leaf temperature and low LWP could be adjusted stomatal close then reduce water consumption and T_r_. Interestingly, we observed no significant difference between CK and DS in Δ, while HY113 showed higher IWUE and water productivity but lower Δ than YLY6 (Fig. 4). The stable Δ shows negative relation with WUE in plants^28,29,32,71^. The inconsistence among these previous studies might be probably due to the sampling location, timing (growth stage) and extent of DS, lead to variation in outcomes^32,71^.

## Conclusion

DS at flowering stage could significantly reduce GY, with decline in SPN and FG. YLY6 showed significant reduction in physiological activities in response to DS, with better recovery capability after DS than HY113. Our results suggest that it is improper to evaluate drought resistance ability only by the performance in response to drought at flowering stage, because the selected susceptible varieties may have better recovery capability after DS than the selected resistant varieties. Thus, better recovery capability is important to keep relatively high grain production under DS conditions.

## Materials and Methods

### Site description

Field experiments were conducted in 2013 and 2014 under a rainproof shelter at Huazhong Agricultural University, Hubei Province, China (30°28′N, 114°21′E) during rice growing season (May to October). The soil type was a clay loam (pH = 5.7) with 18 g kg^−1^ organic matter, 1.4 g kg^−1^ total N, 0.85 mg kg^−1^ total P, and 118.8 mg kg^−1^ total K. The field capacity soil moisture content was 28.5% and bulk density of the soil was 1.2 g cm^−3^. To minimize water infiltration between plots, plastic film was applied on the plot bunds and the plastic film was installed under soil surface with a depth of 15 cm. To prevent water from CK to DS, the main plots were separated with cement concrete (20 cm width). The average data of air temperature during the rice-growing across two study years recorded by temperature and humidity monitoring equipment (TPJ-20, Tuopu Instruments Ltd, Zhejiang, China), installed close to the experimental site, and precipitation was excluded for the rainproof shelter and the open-ended rainproof shelter ensures that rice growth was not affected by high temperature.

### Experiment design

Two water management treatments were compared in a split-plot with four replicates with a plot size of 3 m × 6 m in 2013 and 2014. The main plots were two water treatments, including CK and DS at the flowering stage. The sub-plots were two rice cultivars, “super” hybrid rice of YLY6 and drought tolerance rice of HY113. YLY6 is a two-line hybrid rice variety with characteristics of high yield and high quality and higher drought tolerance and bred by Lixiahe Regional Research of Agricultural Science (Jiangsu province, China), widely cultivated in Hubei Province. HY113 is an indica three-line hybrid upland rice has characteristics of drought resistance and capacity of water saving and bred by Shanghai Agricultural Biological Gene Center (Shanghai province, China). The two rice cultivars showed similar growth periods, which can ensure both genotypes flower at the same time.

To better understand the dynamic change of rice before and after DS, we chose four-time nodes; before DS (BDS), DS, after 2 days of rehydration (ARD 2), after 20 days of rehydration (ARD 20) in 2014 as a supplementary trial. The CK treatment plots were puddled and continuously flooded with 1–3 cm water level during the 15 days after transplanting. Thereafter, the water level was gradually increased to 5–10 cm at the full rice crop development until 2 weeks before harvest. While the DS treatment plots were imposed DS by withholding water at the flowering stage during the 60 days after transplanting, and soil water potential was monitored by soil tensiometer (JX-2, Tuopu Instruments Ltd., Zhejiang, China) with three in each plot for maintaining the soil water potential at −30 ± 5 kPa level at 15 cm depth.

Twenty-day-old seedlings were transplanted at a hill spacing of 13.3 cm × 30 cm with two seedlings per hill on 10 May 2013 and 1 May 2014. N fertilizer was applied at the rate of 180 kg N.ha^−1^; 50% as basal, 20% at the tillering stage and 30% at the panicle initiation stage, respectively. Potassium fertilizer and phosphate fertilizer were applied as basal fertilizer at the rate of 100.5 kg K_2_O ha^−1^ and 100.5 kg P_2_O_5_ ha^−1^, respectively. Agro-chemicals and hand weeded practices were applied to diseases, pests and weed management during the growing season to avoid yield loss.

The recorded parameters are described below.

### Leaf gas exchange measurements and Δ

Leaf gas-exchange measurements of rice were carried out under both CK and DS conditions with LI-6400XT portable photosynthesis measurement system (Li-Cor, Lincoln, NE, USA). The net P_n_ (μmol CO_2_ m^−2^s^−1^), G_s_ (mol m^−2^s^−1^), T_r_, (μmol m^−2^s^−1^) of the flag leaves (fully expanded functional leaves) were determined at the flowering stage during 9:00–11:00 without cloud days when the photosynthetic active radiation (PAR) was greater or equal to 1000 umol m^−2^s^−1^ in the morning which could ensure that maximum value could be detected and relative humidity ranging between 45%-55%, a leaf temperature of 30 °C. Nine flag leaves of rice were measured for each treatment. IWUE was calculated as the ratio of P_n_ to G_s_.

Δ was analyzed in rice leaves at flowering stage and in rice grains at maturity. Rice leaves samples were dried at 105 °C for 1 hour then at 80 °C until a stable weight and rice seeds were dried at 25 °C for 3 months and then grind to milled rice. All the dried samples were ground using ball mill MM401 (Retsch, Germany) and sifted through a 0.5 mm screen. Stable Isotope ratio mass spectrometer (Thermo, Delta V Advantage, Agawam, USA) was applied to determine the carbon isotope discrimination in Crop Physiology and Production Center (CPPC) in Huazhong Agricultural University, Wuhan.

### LWP and Air-leaf temperature gap

At the flowering stage, rice fully expanded functional leaves (flag leaves) were sampled and cut into small pieces and mixed immediately with less than 20 s at 13:00–15:00 in a sunny day and the time chose to sample leaves was determined based on a preliminary study when the lowest LWP was achieved, and LWP were determined using a water potential analyzer (WP4C, Decagon Devices Inc., USA).

Meanwhile, the air temperature (T1) above canopy was automatically recorded by a data logger (TPJ-20, Tuopu Instruments Ltd, Zhejiang, China) which was installed above the top of rice canopy in the center of the plot, and the flag leaf (fully expanded functional leaves) temperature was measured using the UT300A infra-red thermometer (TPJ-20, Tuopu Instruments Ltd, Zhejiang, China) during 13:00 to 15:00 the detector ranged with 1 cm the leaf surface and temperature of flag leaves (T2) were measured with a portable infrared thermometer UT300A (TPJ-20, Tuopu Instruments Ltd, Zhejiang, China), on sunny days. The time chosen to measure leaf temperature was determined based on a preliminary study (data not shown) when the stable air temperature was achieved. The ALTG was calculated by air temperature and leaf temperature difference, as described: ALTG = T1 − T2.

### Harvesting and grain quality measurements

At maturity stage, GY was obtained from a 5-m^2^ area in each plot except board line, and the standard grain moisture content of 14% was applied to yield calculation. What is more, 8 hills were taken from each plot according to the average number of EPs, and each hill was processed separately. Panicles number was recorded from every hill, each panicle was hand-threshed and the unfilled spikelets were separated from filled spikelets through a blower. The EP, TGW, FG were calculated. FG was calculated as the described formula: 100× filled spikelet number/total spikelet number, and the SPN as follow: GY per square meter/(TGW × EP × FG).

After measuring the GY components, grains harvested from each plot were retained and dried at 40 °C in a dryer to keep the water content at 13% before quality measurements. A 125 g sample of rice grains were dehulled by dehusker, milled by a polisher, then separated into broken and unbroken grains, and weighted respectively. Estimate the BRR, HRR, and MRR were expressed as percentages of total (125 g) rice grains. The flatbed scanner WinRHIZO (Microtek, Shanghai, China), SC-E software (Hangzhou, Wanshen Detection Technology Co., Ltd., Hangzhou, China) and professional image analysis software Image J (National Institutes of Health- NIH, USA) were applied to analysis the GS, CH and CHK using milled grains according to Rice Quality Measurement Standards (Ministry of Agriculture, PR China, 1988). The amylose content and alkali spreading value were measured using head grains according to Rice Quality Measurement Standards (Ministry of Agriculture, PR China, 1988, and the protein in milled grains was determined using an infrared grain quality analyzer (TM-1241, Foss Tecator AB, Denmark).

### Statistical analysis

Data were analyzed by analysis of variance (ANOVA) (SAS Institute, 1999) and means were compared based on the least significant difference (LSD) test at the 5% probability level. The correlation among growth characteristics of these two cultivars was measured by using the CORR model in SAS. Principal components analysis (PCA) was performed in CANOCO 5.0 (Microcomputer Power Ithaca, USA; Braak and Smilauer, 2012).
